# Combined effects of biochar and biodegradable mulch film on chromium bioavailability and the agronomic characteristics of tobacco

**DOI:** 10.1038/s41598-024-56973-8

**Published:** 2024-03-22

**Authors:** Yuan Tang, Fumin Zuo, Changhong Li, Qinghai Zhang, Weichang Gao, Jianzhong Cheng

**Affiliations:** 1 School of Public Health, the Key Laboratory of Environmental Pollution Monitoring and Disease Control, Ministry of Education, Guizhou Medical University, Guian New Area, 561113 Guizhou China; 2 Guizhou Academy of Tobacco Science, Guiyang, 550081 Guizhou China; 3 State Key Laboratory of Environmental Geochemistry, Institute of Geochemistry, Chinese Academy of Sciences, Guiyang, 550081 Guizhou China

**Keywords:** Biochar, Biodegradable mulch film, Bioavailability, Chromium, Flue-cured tobacco, Biogeochemistry, Environmental sciences

## Abstract

Biochar (BC) and biodegradable mulch film (BMF) are both commonly used means of production in agriculture. In recent years, most studies have focused on the effects of BC or BMF on soil heavy metal pollution, while they have neglected the combined effects. In this study, a pot experiment was conducted to examine the impacts of BMF, BC, and combined BMF and BC (CMB) on the mobility of chromium (Cr) and the agronomic characteristics of flue-cured tobacco. Compared with the control, BMF, BC, and CMB significantly reduced the concentrations of diethylenetriamine pentaacetic acid (DTPA) extractable Cr in soils by 29.07–29.75%, 45.35–48.54%, and 34.21–37.92%, respectively. In comparison to the application of BMF and BC alone, co-application reduced the availability of Cr in soil via increasing the adsorption of soil Cr and soil enzyme activity, which resulted in the decrease of Cr content and bioconcentration factor and in plants. Moreover, the combined application increased the plant height, stem diameter, leaf area, total root area, root tip number, and root activity of tobacco, which leaded to increase in leaf and root biomass by 11.40–67.01% and 23.91–50.74%, respectively. Therefore, the application of CMB can reduce the heavy metal residues in tobacco leaves and improve tobacco yield and quality.

## Introduction

Chromium (Cr), the seventh most abundant element on Earth^1^, is classified as the first carcinogenic element^2^ and is also considered the fifth of the potentially toxic elements (PTEs)^3^. In industrial production, Cr is usually discharged into the environment through wastewater and waste gas, where it pollutes and polluting water, soil, and crops. With the increase of Cr pollution, soil pH, alkaline hydrolyzed nitrogen, and urease and sucrase activities all decrease^4^. The accumulation of Cr in plants causes stressors that hinder their growth, resulting in decreased crop production and quality as growing time is extended^5,6^. Moreover, Cr can enter the human body through the food chain and seriously endanger human health^7^ due to its carcinogenic, teratogenic, and mutagenic effects on humans and animals^8,9^. Consequently, the effective remediation and usage of Cr-contaminated soil are critical to promoting sustainable land use and ensuring crop production safety and human health.

Currently, several methods have been proposed to remove heavy metals (HMs) from water and soil, including phytoremediation, physical remediation, chemical remediation, and bioremediation^10^. Physical remediation refers to the use of physical engineering measures to remove Cr. Although this method has advantages because it is timely and effective, physical remediation generally requires extensive engineering and investment, and the migration of contaminated soil may cause secondary pollution. In addition, this method is not suitable for remediating large-scale pollution^11^. Chemical remediation of soil refers to the addition of chemical agents to contaminated soil to change the morphology and valence state of Cr, and reduce the fluidity and toxicity of Cr^12^. However, some chemical remediation methods cause the soil to lose its value for cultivation, and may even cause secondary soil pollution. The bioremediation pathways of Cr in soil include adsorption by animals, plants, and microorganisms^13^. Currently, most of the reports on Cr adsorption by microorganisms are concentrated in the laboratory stage, and less attention has been paid to the application of this technology in this field. Recently, the use of biochar (BC) as an environmental sorbent has become one of the most attractive research hotspots due to its advantages, including abundant raw materials, easy preparation, and stable performance^14–16^. In addition, BC plays an important role in decreasing the availability of HMs and plant HMs^17–19^. More importantly, BC can increase crop yields through improving soil fertility and can remediate soil via immobilizing HMs^20,21^. In previous studies, applying BC to contaminated soil in tanneries was found to be an effective way to reduce Cr toxicity and promote plant health and growth^22,23^. In addition, BC demonstrated a comprehensive effect on paddy soil properties, soil microbial communities, soil aggregates, and Cr mobility; improved the soil carbon sequestration capacity; and reduced the accumulation of Cr in rice grains^24^. Therefore, BC as a green environmental adsorbent, has great advantages in remediating heavy metal pollution.

China is the largest tobacco producer in the world, with a tobacco planting area of about 1.08 million hectares and an annual tobacco output of about 2.1 million tons^25^. Mulching is often used as an important way to improve the yield and quality of flue-cured tobacco. However, the long-term and continuous use of plastic mulch results in the deposition of residual plastic film into the soil, culminating in irreversible soil pollution^26–28^. In recent years, to mitigate the negative effects of residual plastic mulch in the soil, the use of biodegradable mulch film (BMF) has increased^29^. The use of BMF reduces soil damage and has a positive effect on soil heavy metal levels. Studies have shown that after 6 weeks of BMF degradation, the soil Cr levels are reduced^30^ and the existence of BMF reduces the bioavailability of metals in some cases^31–34^. However, there are few studies on the effects of combined BC and BMF (CMB) on Cr mobility in a tobacco-planting soil. Therefore, the present study was conducted with the objectives to (i) investigate impacts of the tobacco straw waste derived BC and BMF co-application on reducing soil available Cr and bioaccumulation in different parts of tobacco plants, (ii) the impacts of CMB on soil nutrient and enzyme activities were studied and (iii) the impacts of CMB on the growth and yield of tobacco crop.

## Materials and methods

### Materials

The soils were selected from 0 to 20 cm of the cultivated layer in the tobacco-growing area of Pingba country in Guizhou province, China. The soil type is classified as a yellow loam (Chinese Soil Taxonomy). The basic physico-chemical properties of tobacco-growing soil are as follows: soil pH of 5.99; organic matter content of 45.16 g/kg; and available N, P, and K contents of 187.92, 17.85, and 1121.12 mg/kg, respectively. The BMF is commercially available with homogeneous and dense and without fractures, and its main component is a polybutylene adipate terephthalate (PBAT). The density of BMF was 1.38 g/cm^3^, and thickness was 0.008 mm. The BC was produced made locally using flue-cured tobacco stems as raw material, which was converted into BC at a pyrolysis temperature of 500 °C under anaerobic conditions. Flue-cured tobacco stems was pyrolyzed at 500 °C in a horizontal fixed-bed (internal diameter of 4.5 cm and 24 cm long) reactor, the pyrolysis temperature was increased at a rate of 100 °C /min until the desired temperatures were reached, and then 2 h residence time was applied to perform slow pyrolysis process under 50 L/min nitrogen gas atmosphere, after which cooled down to 500 °C with the continuous flow of nitrogen. Its basic physico-chemical indexes include pH (9.92); conductivity (5.5 µS/cm); solid yields (29.44%); ash content (12.51%).

### Pot experiment

The experimental research and field studies on plants (either cultivated or wild), including the collection of plant material, are comply with relevant institutional, national, and international guidelines and legislation. The Cr-contaminated soil was prepared by mixing 8.0 kg of soil in each pot with a certain volume of Cr(NO_3_)_3_ solution before the experiment. The artificial contaminated soil contained a Cr concentration of 150 mg/kg. Subsequently, the homogeneous mixture was dried naturally, and BC and BMF were added after 30 days. The experiment included four treatments with three replicates as follows: no amendments (CK), biodegradable mulch film (BMF) amendment, biochar (BC) amendment, and combined biodegradable mulch film and biochar (CMB) amendment (Table 1). A total of 28 experimental pots were set up. The soil was air-dried, passed through a sieve with 2-mm mesh size, and mixed with compound fertilizer of flue-cured tobacco (54.55 g per pot and N:P_2_O_5_:K_2_O = 1:1:2.5), and healthy tobacco plant seedlings (Yunyan 87) of the same age and consistent growth were transplanted into each pot. The potted plants were fertilized again (30 g per pot and N:P_2_O_5_:K_2_O = 1:1:2.5) after 30 days. One tobacco seedling was planted in each pot. During the growth of the tobacco, crop cultivation management was consistent with normal production practices, with regular weeding, insect killing, and watering. After 50 days, three repeats were randomly selected for each treatment to be the first crop samples. The remaining four pots from each treatment were the second crop samples, which continued to grow for 100 days.

**Table 1:** Experimental treatment design.

Treatments	Biochar (g)	Biodegradable mulch film (g)	Cr(NO_3_)_3_ (g)
CK	0	0	9.2
BMF	0	4	9.2
BC	4	0	9.2
CMB	2	2	9.2

### Soil sample analysis

#### Soil physicochemical analysis

Soil samples in the incubation experiment were collected every 50 days. The electrical conductivity and soil pH were measured in a soil–water suspension (1:5 ratio, w/v) using a potentiometric method^35^. The soil organic matter content was determined using the potassium dichromate volumetric method^36^. The alkaline hydrolysis diffusion method was used to determine the soil available nitrogen content^37^. The soil available phosphorus content was determined spectrophotometrically using a continuous flow analyzer (SAN +  + , Skalar Analytical B.V., Breda, the Netherlands)^38^. The soil available potassium was extracted with 1.0 M of ammonium acetate for 30 min and the content was determined using flame photometry^37^.

#### Soil Cr analysis

The total Cr content was determined using a microwave digester (Speedwave Xpert, Berghof, Germany). Specifically, samples (0.2 g) were digested with a dup-acid mixture of HF (1 mL) and HNO_3_ (8 mL), and the residual solution was filtered through 0.22-μm membrane filters and diluted to 50 mL^39^. The available Cr was analyzed using the diethylenetriamine pentaacetic acid (DTPA) extraction method and then subjected to inductively coupled plasma-mass spectrometry Agilent 7700 ICP-MS (Agilent Technologies, Santa Clara, CA, USA) analysis with a limit of quantification of 0.03 μg/L.

#### Soil enzyme activity analysis

The catalase activity was determined by back-titrating the residual hydrogen peroxide (H_2_O_2_) with potassium permanganate (KMnO_4_)^40^. The urease activity of the soil was determined using the sodium phenolate-sodium hypochlorite colorimetric method^41^. The soil fluorescein diacetate hydrolase activity was determined colorimetrically using fluorescein diacetate as substrate at 490 nm^42^.

### Plant analysis

The plants were thoroughly washed with deionized water and divided into three parts (the root, stem, and leaf). The Cr concentration in plant organs was determined by applying acidic-oxidant microwave digestion with a mixture of Suprapur^®^ grade HNO_3_ 65% and H_2_O_2_ 30% 7/2 (v/v), followed by ICP-MS analysis^43^. A root scanning analysis system (WinRHIZO, Regent Instrument, Canada) was used to analyze root biological properties such as the root length, root surface area, root volume, average root diameter, and root tip number. Root activity was determined in 0.2 g root samples using the 2, 3, 5-triphenyltetrazolium chloride (TTC) method^44^. The survey items included the plant height, stem circumference, effective leaf number, and leaf area. The leaf area was calculated as d = (L + 2 W)/3, where d, L, and W represent the leaf area, maximum leaf length, and maximum leaf width, respectively. The number of leaves was counted, and the plant height was measured. The plants were separated into roots, stems, and leaves, and the fresh biomass of each component was weighed^45^. Each obtained whole tobacco plant sample was placed at 115 °C for 25 min to inactivate the cells and then dried at 55 °C and weighed to obtain the dry matter mass of the tobacco plant^44^.

### Quality control and data analyses

For accuracy and precision of data, the certified standards were used in each batch of plant extraction. All the treatments were conducted in triplicate. Data were presented as the means with standard deviations. Statistical analysis of the experimental data was performed using one-way analysis of variance (ANOVA) with the SPSS 22.0 statistical software package (SPSS Inc., Chicago, IL, USA). The means of the data were compared based on the least significant difference (Duncan) test at a 5% significance level^46^. The bio-concentration factor (BCF) and translocation factor (TF) were calculated as follows:

$$\text{BCF} = \frac{{\text{C}}_{{\text{Cr}} \, {\text{in}} \, {\text{plant}} \, {\text{tissue}}}}{{\text{C}}_{{\text{Cr}} \, {\text{in}} \, {\text{soil}}}}\text{,}$$

$$\text{TF} = \frac{{\text{C}}_{\text{Cr in shoot}}}{{\text{C}}_{\text{Cr in root}}}\text{,}$$

where C_Cr_ in plant tissue is the concentration of Cr in the roots, stems, and leaves, and C_Cr_ in soil is the concentration of Cr in soil. C_Cr_ in shoot is the concentration of Cr in leaves and stems and C_Cr_ in root is the concentration of Cr in roots.

## Results

### Changes of total Cr content and DTPA-extractable Cr in soil

**Figure 1:**
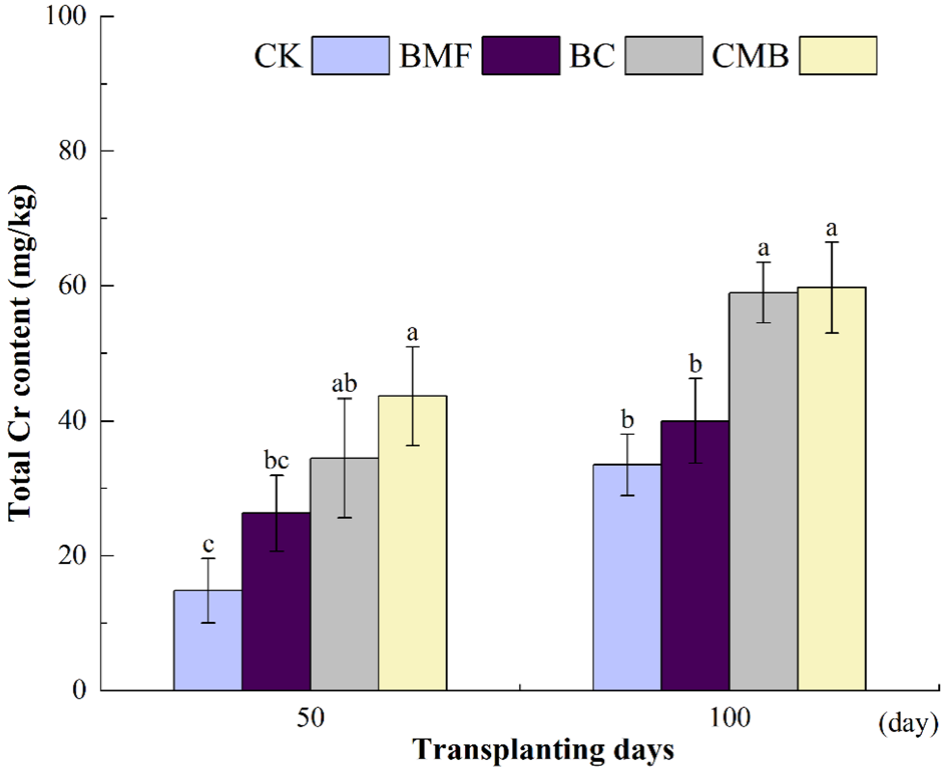
Impacts of BMF, BC and CMB treatments on the total Cr concentration in soil. The different letters indicate significant difference at p ≤ 0.05. No amendments (CK), biodegradable mulch film amendment (BMF), biochar amendment (BC), combined biochar and biodegradable mulch film amendment (CMB).

Compared with the CK treatment, the total Cr contents in the BC and CMB treatments on the 50th day of tobacco transplantation increased by 132.58% and 194.38%, respectively (Fig. 1). On the 100th day of tobacco transplantation, the total Cr contents in the BC and CMB treatments increased by 76.12% and 78.36%, respectively. There were significant differences in the total Cr content between the CMB treatment and CK at both day 50 and day 100. Moreover, the combined application of BMF and BC significantly improved the soil Cr content compared to the application of BMF and BC alone.

**Figure 2:**
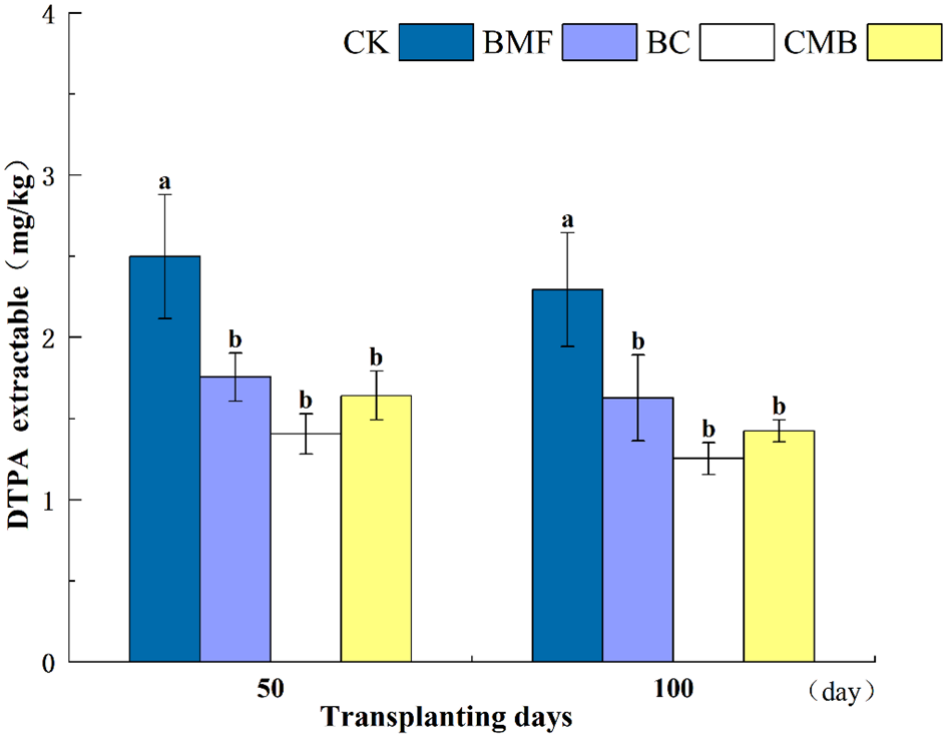
Impacts of BMF, BC and CMB treatments on the diethylenetriamine pentaacetic acid (DTPA) extractable Cr concentration in soil, the different letters indicate significant difference at p ≤ 0.05.

In comparison with CK, the DTPA-extractable Cr significantly decreased in soil treated with BC, BMF, and CMB at both 50 and 100 days (Fig. 2), and the concentration of DTPA-extractable Cr in all treatments decreased gradually with incubation time. Compared with CK, the DTPA-extractable Cr contents in the BMF, BC, and CMB treatments were significantly lower at 50 days by 29.75%, 48.54%, and 34.21%, respectively. At 100 days, the decrease in DTPA-extractable Cr was more pronounced, and the concentrations of DTPA-extractable Cr in BMF, BC, and CMB were significantly reduced by 29.07%, 45.35%, and 37.92%, respectively. It was observed that CMB significantly decreased the concentration of DTPA-extractable Cr with incubation time.

### Changes of enzyme activity in soil

**Figure 3:**
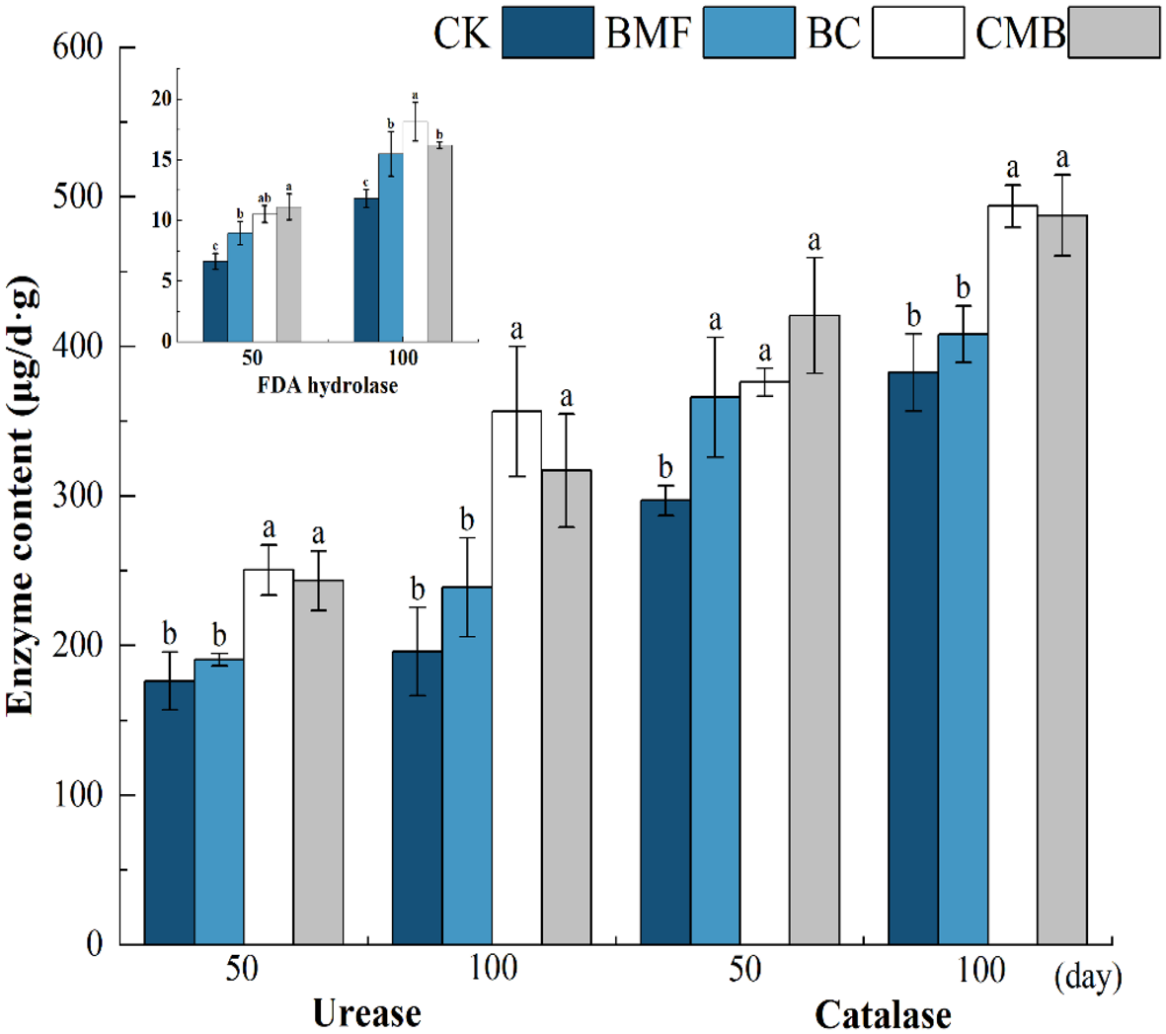
Impacts of BMF, BC, and CMB treatments on soil enzyme activity. The different letters indicate significant difference at p ≤ 0.05.

Compared with CK, the BC and CMB treatments significantly increased the urease activities of soil, and soil urease activities in the BC and CMB treatments increased by 41.94% and 37.92% at 50 days, respectively, and by 81.98% and 61.78% at 100 days, respectively (Fig. 3). Of the four treatments, BC had the highest urease activity, followed by CMB, BMF, and CK, and the soil urease activity in all treatments increased with incubation time. Compared with CK, the catalase activities in the BMF, BC, and CMB treatments significantly increased by 23.19%, 26.60%, and 41.60% at 50 days, respectively, and by 6.68%, 29.07%, and 27.43% at 100 days, respectively. Compared with CK, the soil FDA hydrolase activities after 50 days were significantly increased by 35.35%, 59.06%, and 67.98% in the BMF, BC, and CMB treatments, respectively. Compared with the CK treatment, the soil FDA hydrolase activity was significantly increased by 30.94%, 53.55%, and 37.00% in the BMF, BC, and CMB treatments on the 100th day of tobacco transplantation, respectively. In a word, the results show that BMF, BC, and CMB can significantly increase soil enzyme activity with incubation time.

### Changes in the Cr content, BCF, and TF in flue-cured tobacco

**Figure 4:**
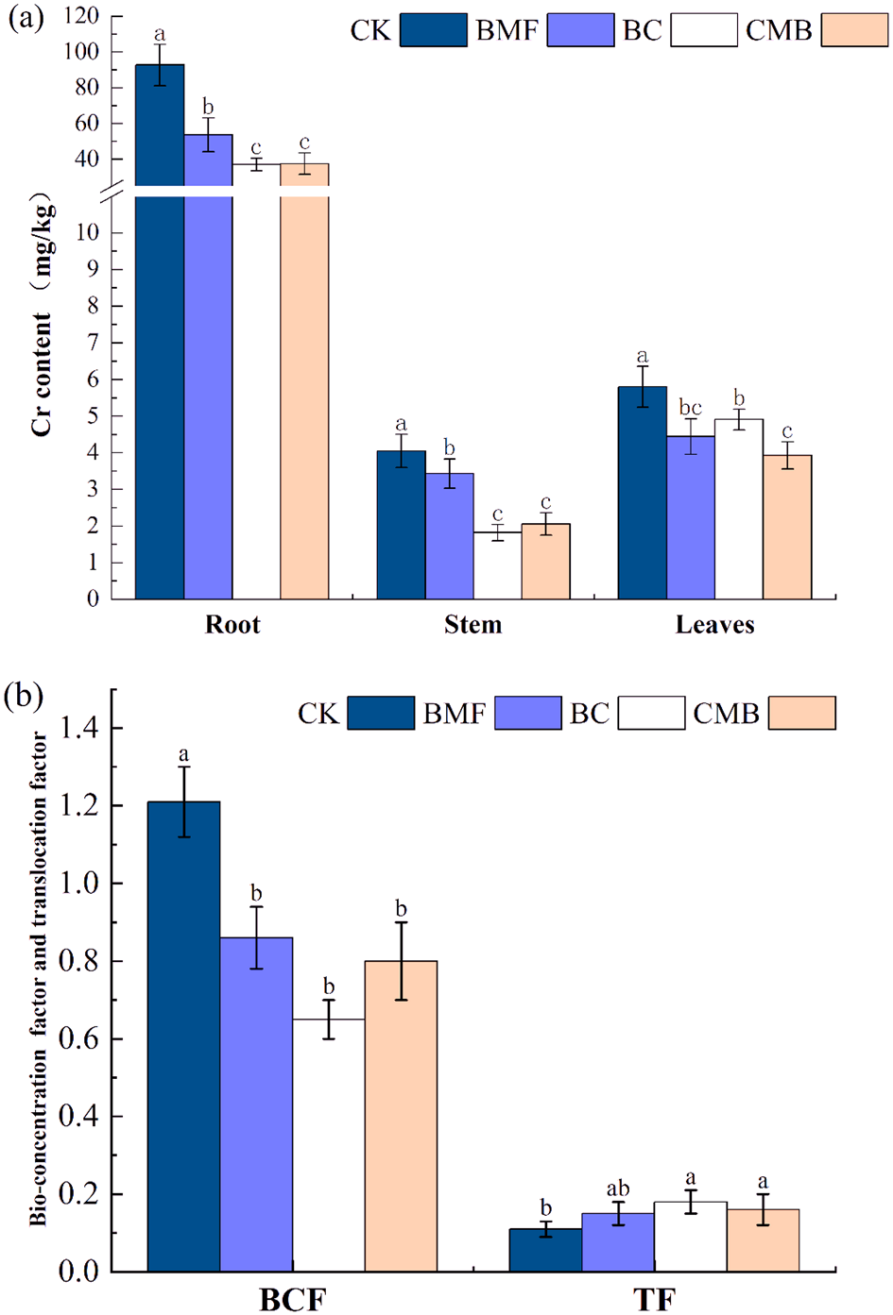
Impacts of BMF, BC and CMB treatments on Cr contents in the roots, stems, and leaves of tobacco (**a**) and the bio-concentration factor (BCF) and translocation factor (TF) values of Cr in tobacco (**b**). The different letters indicate significant difference at p ≤ 0.05.

Compared with CK, the Cr contents in roots were significantly reduced by 42.05%, 60.02%, and 59.48% in the BMF, BC, and CMB treatments, respectively (Fig. 4a). However, there were no significant differences between the BMF, BC, and CMB treatments. Compared with CK, the Cr contents in stems were significantly reduced by 15.25%, 55.04%, and 49.16% in the BMF, BC, and CMB treatments, respectively, and the stem Cr content in CMB was significantly different from that in BMF but not significantly different from that in BC. In comparison with CK, the Cr contents in the leaves of BMF, BC, and CMB treatment samples were significantly reduced by 23.39%, 15.38%, and 32.23%, respectively, while the differences between CMB and BMF were not significant, and the differences between CMB and BC were significant. Upon the addition of BMF, BC, and CMB, in comparison with CK, the BCFs were significantly decreased by 57.67%, 73.33%, and 77.67% (Fig. 4b), Among the four groups, CMB had the lowest BCF respectively, while the TFs were significantly increased in BC and CMB.

### Effects of different treatments on the root activity, morphology, and agronomic characteristics of tobacco plants

**Figure 5:**
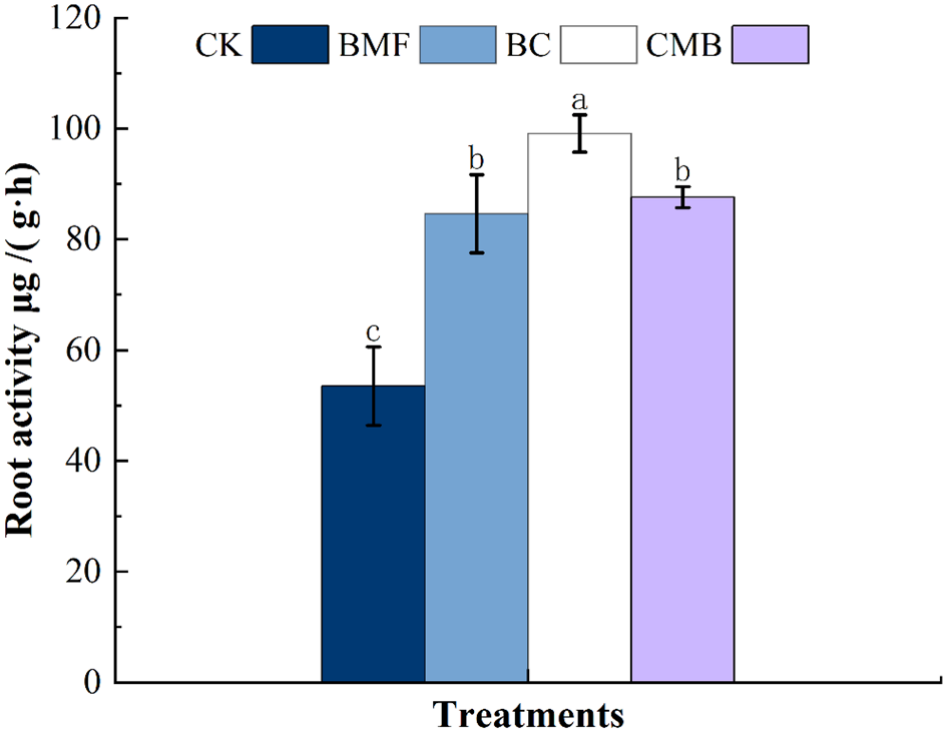
Impacts of BMF, BC and CMB treatments on the tobacco root activity. The different letters indicate significant difference at p ≤ 0.05.

Compared with CK, the root activity of flue-cured tobacco increased by 58.13%, 85.20%, and 63.69% in BMF, BC, and CMB, respectively (Fig. 5). Nevertheless, there were no significant differences between BMF, BC, and CMB. Compared with CK, the root length, root projected area, root surface area, and root volume in the BC treatment increased by 57.40%, 65.68%, 69.41%, and 84.13%, respectively (Table 2); the corresponding values in treatment CMB increased by 46.31%, 37.77%, 59.84%, and 50.88%, respectively; and the corresponding values in treatment BMF increased by 16.00%, 10.74%, 38.74%, and 29.22%, respectively. There were no significant differences in root length, root projection area, root surface area, or root volume area between BMF and CMB, but root volume was significantly different between BC and CMB.

**Table 2:** Impacts of BMF, BC, and CMB treatments on the root morphology in tobacco.

Treatments	Root length/(cm)	Projection area/(cm^2^)	Surface area/(cm^2^)	Root volume/(cm^3^)
CK	1568.00 ± 344.78b	94.16 ± 21.26c	258.75 ± 35.29b	3.97 ± 0.62c
BMF	1818.92 ± 513.79ab	104.27 ± 21.30bc	358.99 ± 95.95ab	5.13 ± 1.15bc
BC	2468.00 ± 189.53a	156.00 ± 8.79a	438.35 ± 79.99a	7.31 ± 1.52a
CMB	2294.20 ± 718.60ab	129.72 ± 25.52ab	413.59 ± 89.77a	5.99 ± 1.25ab

The different letters indicate significant difference at p ≤ 0.05. No amendments (CK), biodegradable mulch film amendment (BMF), biochar amendment (BC), combined biochar and biodegradable mulch film amendment (CMB).

**Figure 6:**
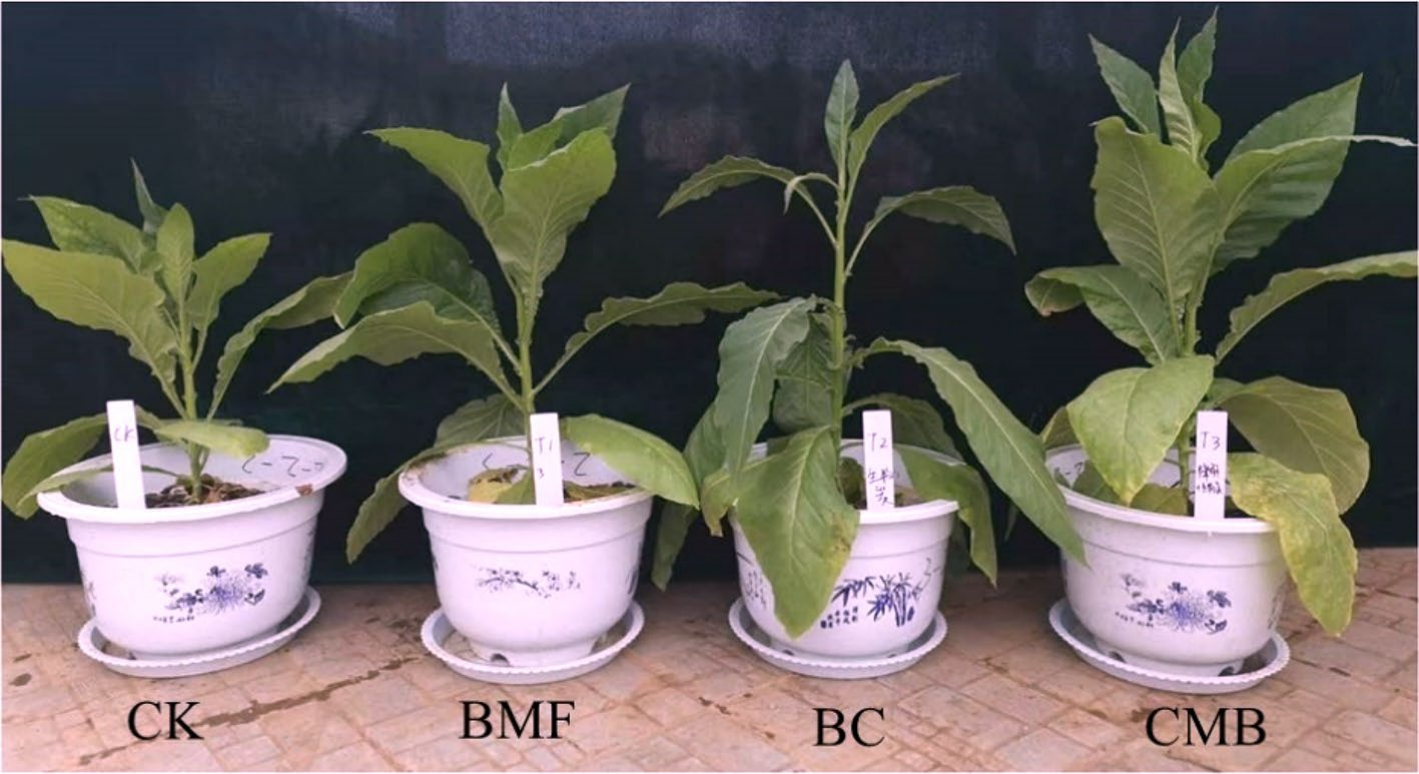
Impacts of BMF, BC, and CMB on the growth of flue-cured tobacco at 100 days.

Compared with CK, the productive leaf number, leaf area, and plant height of BC and CMB were significantly increased in two transplantation periods (Table 3, Fig. 6). At day 50, compared with CK, the productive leaf number of the BC and CMB treatments significantly increased by 30.68% and 26.87%, respectively. The leaf area of the BC and CMB treatments significantly increased by 60.91% and 60.43%, respectively. The stem biomass of the BC and CMB treatments significantly increased by 100.00% and 61.54%, respectively. The productive leaf number, leaf area, plant height, and stem biomass were significantly higher in CMB than in BMF, but the difference between CMB and BC was not significant. At day 100, compared with CK, the leaf areas of the BC and CMB treatments were significantly increased by 57.90% and 68.7%, respectively. The stem biomass of the BC and CMB treatments significantly increased by 135.48% and 118.82%, respectively, and the leaf biomass significantly increased by 80.67% and 67.01%, respectively. The plant height, stem girth, leaf area, stem biomass, and leaf biomass of the CMB treatment were significantly higher than those of BMF, but the difference between CMB and BC was not significant.

**Table 3:** Impacts of BMF, BC, and CMB treatments on the agronomic traits and biomass of tobacco.

Time	Treatments	Productive leaf number	Leaf area (cm^2^)	Plant height (cm)	Stem girth (cm)	Root biomass (g)	Stem biomass (g)	Leaf biomass (g)
50d	CK	8.67 ± 0.58b	115.10 ± 6.97b	8.37 ± 0.25b	5.14 ± 3.01b	0.31 ± 0.04b	0.13 ± 0.00b	1.93 ± 0.09ab
	BMF	9.33 ± 1.15b	118.57 ± 9.72b	9.20 ± 0.36b	5.15 ± 0.13b	0.47 ± 0.02a	0.19 ± 0.26ab	1.70 ± 0.17b
	BC	11.33 ± 0.58a	185.21 ± 27.60a	10.26 ± 0.89a	5.72 ± 0.25a	0.58 ± 0.06a	0.26 ± 0.69a	2.33 ± 0.40a
	CMB	11.00 ± 0.00a	184.65 ± 28.87a	10.17 ± 0.91a	5.37 ± 0.40ab	0.58 ± 0.02a	0.21 ± 0.05a	2.15 ± 0.30ab
100d	CK	14.75 ± 0.96b	272.69 ± 75.55b	32.62 ± 2.70b	7.33 ± 0.25c	2.03 ± 0.58a	1.86 ± 0.52b	7.76 ± 1.44b
	BMF	16.75 ± 0.96a	304.21 ± 67.95b	37.55 ± 5.20ab	8.28 ± 0.46bc	2.35 ± 0.51a	2.76 ± 0.86ab	9.40 ± 1.61ab
	BC	17.50 ± 0.58a	430.59 ± 51.52a	43.95 ± 6.37a	9.95 ± 1.39ab	2.76 ± 0.46a	4.38 ± 0.97a	14.02 ± 1.90a
	CMB	18.50 ± 1.73a	460.04 ± 106.93a	42.95 ± 6.07a	9.52 ± 1.37a	3.06 ± 1.31a	4.07 ± 1.96a	12.96 ± 3.26a

The different letters indicate significant difference at p ≤ 0.05.

## Discussion

### Effects of CMB on soil Cr availability

After the addition of BMF, BC, and CMB, the Cr contents in the soil of treatments BMF, BC, and CMB increased significantly, while the content of DTPA-extractable Cr decreased significantly (Figs. 1 and 2). Similar results were found by Refs.^47,48^ and^49^. According to previous research, BC is effective in remediating Cr contamination. For example, BC lowered the DTPA-extractable Cr in spinach-planting soil^46^, BC was shown to reduce the Cr availability of soil contaminated by tannery waste^50^, and Zaman et al.^23^ reported that as wheat straw BC content increased, the content of Cr (DTPA-extracted) in soil fell significantly. Furthermore, the addition of BMF can reduce the bioavailability of HMs in soil^31,34,51^. For example, Li et al. found that after 6 weeks of degradation, BMF lowered the heavy metal contents (Cr, Ba, As, and Pb) in soil^30^, and Li et al.^51^ showed that the bioavailability ratio of Cd to As decreased after applying BMF, indicating that BMF had a significant effect on Cr reduction. Most importantly, Cr was significantly decreased when BC and BMF were administered in combination, which was consistent with Li et al. ’s^52^ finding that the addition of microplastics (MPs) to sludge BC could reduce the availability of Cr and Pb, and with the increase of MPs, the Cr leaching content further decreased. Then, all of these statements are consistent with our research.

The reduction in Cr availability in CMB-treated soils may be attributed to the adsorption of HMs by BC. This is due to the high porosity, larger surface area, cation exchange capacity, and numerous negative surface functional groups present in BC, which play a crucial role in reducing heavy metal mobility through adsorption and precipitation^53,54^. Moreover, the application of BC to Cr-contaminated soils increases the organic matter content of the soil, which can facilitate Cr adsorption^55,56^. In this study, compared to the control, BC increased the soil organic matter content by 9.02%. Another reason for the reduced Cr availability is the adsorption of HMs by BMF. It has been reported that MPs exhibit a unique property of adsorbing environmental contaminants, such as HMs^57^. This is likely due to electrostatic interactions between the negatively charged MPs and the positively charged anodic pole of HMs. In addition, during the degradation of BMF in soil, more functional groups that can adsorb and interact with HMs are exposed, such as benzene rings, hydroxyl groups, and carboxyl groups^51^. Moreover, it has been reported that BC enhances the adhesion of soil particles on the surface of MPs^58^, because MPs are trapped on the surface of BC during adsorption, metal oxide nanoparticles enhance adhesion through interaction with MPs^59^, and the metal-O-PS bonds formed between microplastic particles and the BC surface contribute to the adsorption of HMs in soil^59,60^. Therefore, the combined application of BC and BMF reduced the biological effectiveness of soil Cr more effectively than solo applications.

### Effects of CMB on the agronomic characteristics of flue-cured tobacco

The addition of CMB not only had a significant effect on reducing soil Cr but also had a positive effect on the growth of flue-cured tobacco. The addition of CMB significantly promoted the root biological traits and agronomic characteristics of flue-cured tobacco (Tables 2 and 3, Fig. 6). Consistent with the findings of the present study, BC has been found to exert beneficial effects on crop growth. For example, the development of spinach roots and stems in Cr-contaminated soil increased after the addition of BC^46^. Similarly, Bashir et al. concluded that the application of BC to Cr- or Cd-contaminated soil greatly increased plant height^61^, while the application of wheat straw BC to Cr-contaminated soil significantly enhanced tomato plant biomass^23^. Furthermore, BMF also has a positive effect on the growth of crops, many studies have been conducted in which the application of BMF to maize^62–64^ and cotton increased yields^65^. This suggests that the addition of CMB can also improve crop growth.

One of the reasons why CMB promotes the growth of flue-cured tobacco is that the improvement of plant growth may depend on the adsorption of soil HMs onto BC and BMF. In the present study, after adding CMB, compared to CK, the bioavailability of Cr in soil was significantly reduced by 37%, and the root, stem, and leaf Cr concentrations in plants were correspondingly reduced by 59.48%, 49.15%, and 32.21%, respectively, which led to a decrease in the transfer and toxicity of Cr to plants^4^, and promoted plant growth. The favorable influence of BC and BMF on soil qualities was another reason why CMB improved the growth of flue-cured tobacco. In this study, it was observed that the addition of BC increased the soil urease activities, catalase activities, soil FDA hydrolase activities, and soil organic matter content by 81.98%, 29.07%, 53.55%, and 9.02% respectively, compared to the CK treatment, which led to an optimized tobacco growth environment^66,67^ and improved crop growth. Moreover, BMF also significantly increased catalase activities and FDA hydrolase activities by 23.19% and 30.96% respectively, which was consistent with previous findings that BMF improved soil urease and soil catalase activities^68^, thereby improving plant growth. After degradation, the residual plastic film fragmented, thinned, become brittle, and clung to the surface, where it continued to play a role in increasing temperature and preserving soil moisture^68^, thus promoting the growth of tobacco. Therefore, CMB can been added to soil to promote the growth of flue-cured tobacco.

## Conclusions

The combination of BMF and BC clearly reduced the Cr bioavailability in tobacco-growing soil. The CMB treatment dramatically decreased the tobacco Cr contents and enhanced the tobacco biomass through boosting soil enzyme activity and reducing Cr toxicity. Compared to CK, the bioavailability of soil Cr in the CMB treatment decreased by 34.21–37.92%; the urease activities, catalase activities, and FDA hydrolase activities significantly increased by 37.92–61.78%, 27.43–41.60%, and 37.06–67.98%, respectively; and the stem and leaf biomass of tobacco increased by 118.82% and 67.01%, respectively. These results showed that BC alone or combined with BMF can reduce the Cr bioavailability of soil and Cr accumulation in tobacco leaves, which is beneficial to food safety and human health. However, the results of this study are based on pot experiments, and a large field trial is required to verify the combined effects of BC and BMF on Cr bioavailability and the agronomic characteristics of tobacco.

## Data Availability

The data that support the findings of this study are available from the corresponding author upon reasonable request.
